# Supplementary material for: Deconvolution-based cell-type specific DNA methylation-wide and transcriptome-wide association studies identify risk CpG sites and genes associated with colorectal cancer risk
Source: medRxiv. 2026 Jun 12:2026.06.11.26355460. Preprint. [Version 1] doi: 10.64898/2026.06.11.26355460 (PMC13278294; doi:10.64898/2026.06.11.26355460)
Supplement: Supplementary file 1 [file NIHPP2026.06.11.26355460V1-supplement-1.pdf]

medRxiv preprint doi: https://doi.org/10.64898/2026.06.11.26355460; this version posted June 12, 2026. The copyright holder for this preprint (which was not certified by peer review) is the author/funder, who has granted medRxiv a license to display the preprint in perpetuity.
It is made available under a CC-BY 4.0 International license .

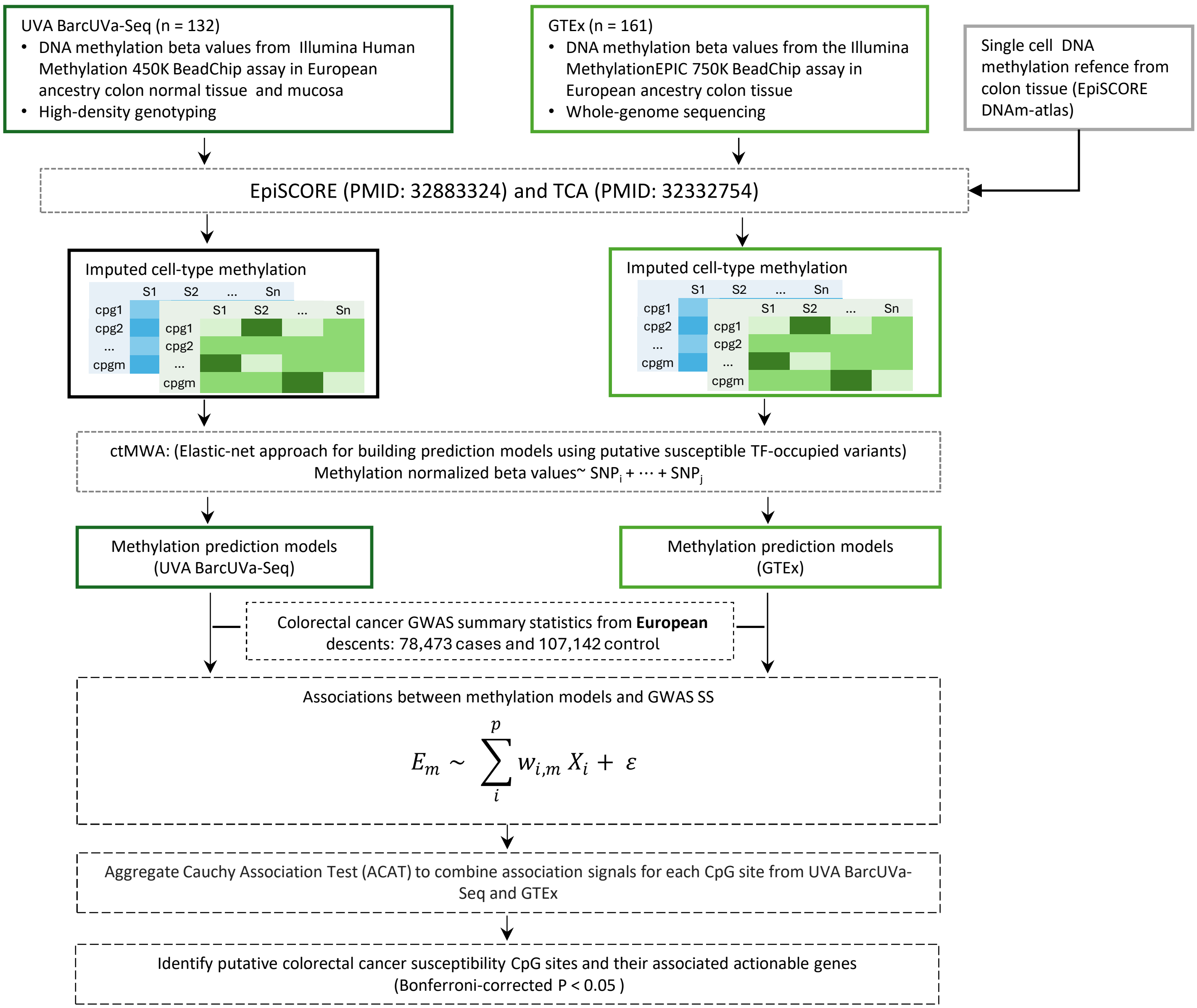

medRxiv preprint doi: https://doi.org/10.64898/2026.06.11.26355460; this version posted June 12, 2026. The copyright holder for this preprint (which was not certified by peer review) is the author/funder, who has granted medRxiv a license to display the preprint in perpetuity.
It is made available under a CC-BY 4.0 International license .

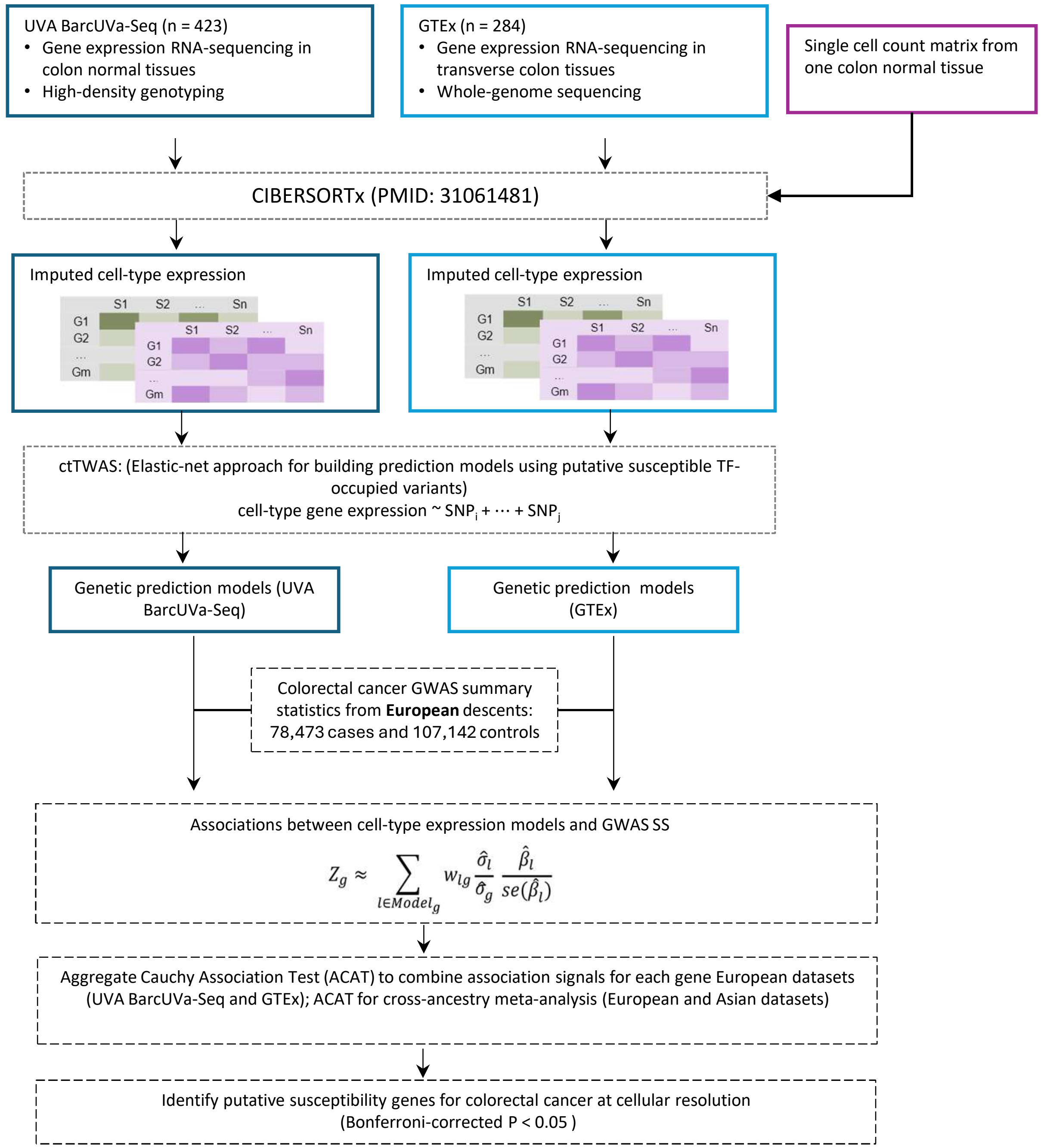

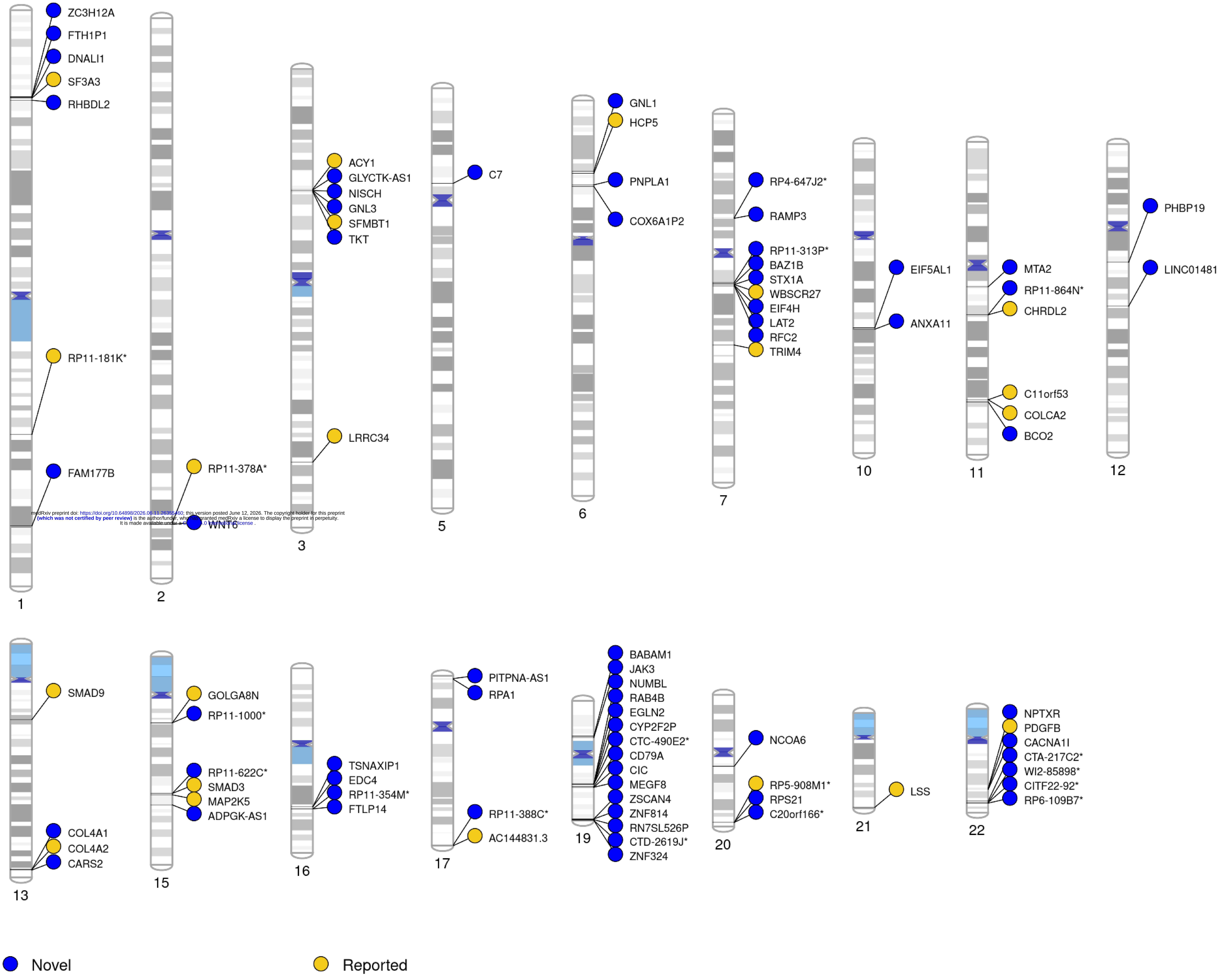

medRxiv preprint doi: https://doi.org/10.64898/2026.06.11.26355460; this version posted June 12, 2026. The copyright holder for this preprint (which was not certified by peer review) is the author/funder, who has granted medRxiv a license to display the preprint in perpetuity.
It is made available under a CC-BY 4.0 International license .
